# No evidence of Fabry disease in a patient with the new p.Met70Val GLA gene variant

**DOI:** 10.1002/mgg3.2390

**Published:** 2024-06-19

**Authors:** Irene Capelli, Roberta Di Costanzo, Valeria Aiello, Sarah Lerario, Paola De Giovanni, Marcello Montevecchi, Davide Cerretani, Vincenzo Donadio, Gaetano La Manna, Renzo Mignani

**Affiliations:** ^1^ Nephrology, Dialysis and Renal Transplant Unit IRCCS Azienda Ospedaliero‐Universitaria di Bologna Bologna Italy; ^2^ Department of Medical and Surgical Sciences (DIMEC) Alma Mater Studiorum University of Bologna Bologna Italy; ^3^ Department of Nephrology Infermi Hospital Rimini Italy; ^4^ IRCCS Istituto delle Scienze Neurologiche di Bologna UOC Clinica Neurologica Bologna Italy

**Keywords:** Fabry disease, pathogenicity, variant, variant of unknown significant

## Abstract

**Background:**

Fabry disease (FD) is a rare X‐linked lysosomal storage disorder caused by variants in GLA gene leading to deficient α‐galactosidase A enzyme activity. This deficiency leads to the accumulation of glycosphingolipids, particularly globotriaosylceramide (Gb3), in various tissues and organs, which can result in life‐threatening complications. The clinical presentation of the disease can vary from the “classic” phenotype with pediatric onset and multi‐organ involvement to the “later‐onset” phenotype, which presents with predominantly cardiac symptoms. In recent years, advances in screening studies have led to the identification of an increasing number of variants of unknown significance that have not yet been described, and whose pathogenic role remains undetermined.

**Methods:**

In this clinical report, we describe the case of an asymptomatic adult female who was found to have a new variant of unknown significance, p.Met70Val. Given the unknown pathogenic role of this variant, a thorough analysis of the potential organ involvement was conducted. The clinical data were analyzed retrospectively.

**Results:**

The analysis revealed that there were no signs of significant organ involvement, and the benignity of the variant was confirmed.

**Conclusion:**

This case underscores the importance of a comprehensive evaluation of new variants of unknown significance to establish their pathogenicity accurately.

## Graphical abstract

In this case report, we describe the case of an asymptomatic adult female who was found to have a new variant of unknown significance, p.Met70Val. Given the unknown pathogenic role of this variant, a thorough analysis of the potential organ involvement was conducted. The analysis revealed that there were no signs of significant organ involvement, and the benignity of the mutation was confirmed. This case underscores the importance of a comprehensive evaluation of new variants of unknown significance to establish their pathogenicity accurately.

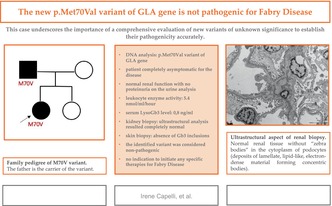

## 1 INTRODUCTION

Fabry disease (FD, OMIM 301500) is an inherited storage disorder that is caused by variants in the GLA gene, located at Xq22. This gene is responsible for encoding the **α**‐galactosidase A enzyme, which plays a crucial role in breaking down glycosphingolipids. When this enzyme is deficient or nonfunctional, there is an accumulation of glycosphingolipids, such as globotriaosylceramide (Gb3), in multiple cell types, tissues, and organs.

The clinical presentation of FD is highly variable, with phenotypes ranging from classic to later‐onset forms. Classic form affected patients (mostly males) exhibit low or absent **α**‐Gal A activity and typically develop the disease in childhood with multi‐organ involvement, including vascular cutaneous lesions (angiokeratomas), sweating abnormalities (anhidrosis, hypohidrosis, and rarely hyperhidrosis), acroparesthesia at the extremities (periodic crises of severe pain in the extremities), characteristic corneal and lenticular opacities (cornea verticillata), and proteinuria; they can also manifest abdominal pain, nausea, or diarrhea. In their transition to adolescence and adulthood, they can manifest renal and cardiac involvement, in addition to cerebrovascular complications. Gradual deterioration of renal function to end‐stage renal disease (ESRD) usually occurs in men in the third to fifth decade.

On the other hand, in a high percentage of patients, the residual leukocyte **α**‐Gal A activity is above 10% but no more than 30%, and symptoms onset occurs in the fourth‐to‐fifth decades of life (Germain et al., 2022). In these late‐onset phenotypes, the progression and the severity of the disease are variable, with symptoms mainly represented by cardiac involvement, which can be severe and may require interventions.

By contrast, heterozygous females may be asymptomatic or have milder symptoms at a later age of onset than males, but not rarely they can also have symptoms as severe as those observed in male patients with the classic phenotype. Several factors can be responsible for this phenotypic variability among heterozygotes, including skewed X‐chromosome inactivation (Germain, 2010).

Currently, the most effective and dependable approach to diagnose FD in males is the demonstration of markedly decreased α‐GalA activity in gold standard samples, such as leukocytes or fibroblasts, which can be confirmed through GLA variant analysis (Desnick et al., 2001). On the other hand, due to random X‐chromosomal inactivation, at least 40% of GLA‐variant‐confirmed female heterozygotes have normal or slightly decreased α‐GalA activity, requiring GLA sequencing to confirm heterozygosity (Desnick et al., 2003; Echevarria et al., 2016; Linthorst et al., 2008).

To date, more than 1000 different GLA variants have been identified in Fabry patients (http://fabry‐database.org). However, the correlation of specific variants with the clinical phenotype is far from straightforward. Most of the pathogenic GLA variants are private, occurring in a single or few families; intrafamilial phenotypic variability has been observed, complicating the study of genotype–phenotype correlations (Mignani et al., 2017). Moreover, recent high‐throughput next‐generation sequencing (NGS)‐based screening programs in high‐risk populations and newborns have identified several novel GLA genotypes, including some variants of unknown significance (VUS), which have not been previously described or investigated.

In these variants, the frequent recognition of nonspecific FD symptoms presents a challenge for physicians attempting to interpret the clinical relevance of a VUS (Germain et al., 2022; Ortiz et al., 2018; Smid et al., 2015). Understanding and interpreting variant pathogenicity is the key to accurate prevalence estimation, diagnosis, and management of FD. Therefore, the identification of markers or patterns that can discriminate the pathogenic role of the variants represents the main challenge in VUS. In this way, kidney biopsy analyzed by electron microscopy is the most important tool able to interpret the pathogenicity of a VUS. Most of these variants are likely benign or polymorphisms, as there is no published evidence of increased LysoGb3 level or lysosomal substrate accumulation in the tissues expressing them. In other cases, the biochemical and histologic patterns may set out VUS pathogenicity (Schiffmann et al., 2016).

In this study, we present the case of a young asymptomatic female with a new variant of the GLA gene, where a thorough diagnostic clinical work‐up confirmed that the variant was more likely benign for FD, providing valuable insights into understanding the clinical implications of this specific genetic variant.

## 2 DETAILED CASE DESCRIPTION

Our patient was a 41‐year‐old female when she was admitted to our referral center for FD for a clinical evaluation. Her medical history revealed a previous diagnosis of Hashimoto's thyroiditis at the age of 18, which was temporarily treated with levothyroxine. At the age of 32 years, she experienced vocal cord paresis which resolved with steroid treatment. In 2014, she underwent a cerebral magnetic resonance imaging (MRI) due to persistent dizziness, which revealed mild and nonspecific white matter hyperintensity. Subsequently, she was investigated for FD: the patient's leukocyte enzyme activity was within normal range (5.4 nmol/mg/h; reference values >3), as well as the serum LysoGb3 level (0.8 ng/mL; reference values <1.8), while a DNA analysis demonstrated the presence of a new variant, c.208A>G p.Met70Val (GLA: NM_000169.3), which had not yet been described. Notably, the patient did not exhibit common symptoms associated with FD, she never suffered from pain or paresthesias nor showed evidence of angiokeratomas and cornea verticillate. Further investigations, including blood and urine tests, revealed normal renal function (serum creatinine: 0.7 mg/dL; estimated glomerular filtration rate: 95 mL/min/1.73 m^2^) with no proteinuria on the urine analysis, while the urine sediment was clear. The electrocardiogram showed a sinus rhythm and normal PR interval, and, on the echocardiogram, the interventricular septum thickness was 8 mm, the posterior wall thickness was 8 mm, and the left ventricular mass was 43 g/m^2^. A recent cardiac magnetic resonance showed normal results, with the absence of late enhancement and T1 mapping within the normal range. In July 2019, the patient started the Enzyme Replacement Therapy (ERT) with agalsidase alfa at the standard dose of 0.2 mg/kg e.o.w and with a standard infusion schedule (40 min infusion duration). The decision to initiate ERT was made by the initial diagnostic center, probably because they considered the central neurological symptoms to be related to the variant. However, at the third infusion, she complained of abdominal pain, nausea, and fever, which were attributed to the agalsidase treatment. Consequently, the treatment was interrupted, and the symptoms resolved.

**FIGURE 1:**
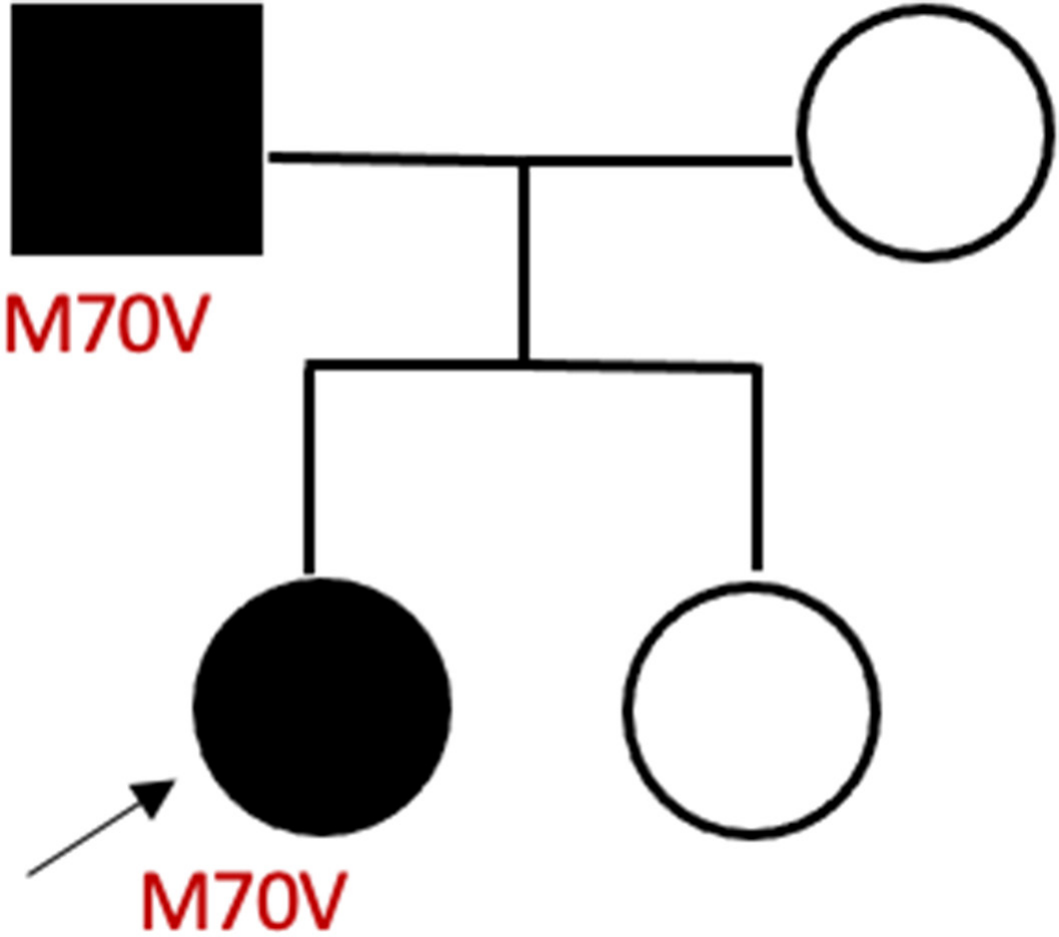
Family pedigree of M70V variant. The father is the carrier of the variant.

In 2020, the patient underwent further evaluation at our referral center, with a focus on the possibility of re‐challenging ERT. Initially, due to the absence of the family screening, which had not been performed previously at the discretion of the clinicians of the initial diagnostic center, enzyme and genotype studies were performed on the patient's parents, resulting in the father being the carrier of the variant (Figure 1). His clinical history only revealed a long history of hypertension and an episode of atrial fibrillation that resolved with electric cardioversion. However, his leukocyte enzyme activity was within the normal range (7.1 nmol/mg/h; reference values >3). Consequently, no further analysis was required.

**FIGURE 2:**
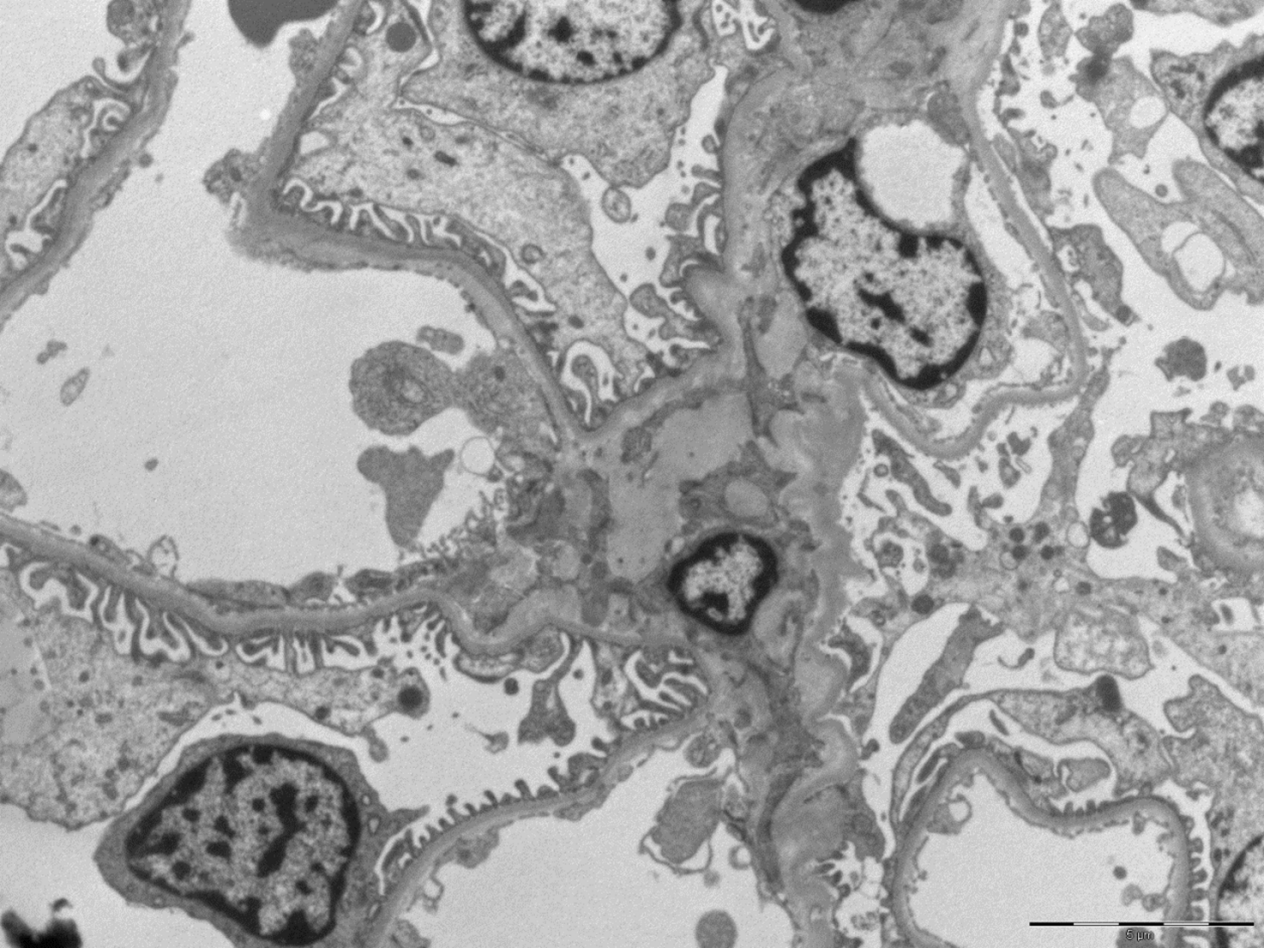
Ultrastructural aspect of renal biopsy. Normal renal tissue without “zebra bodies” in the cytoplasm of podocytes (deposits of lamellate, lipid‐like, electron‐dense materials forming concentric bodies).

**FIGURE 3:**
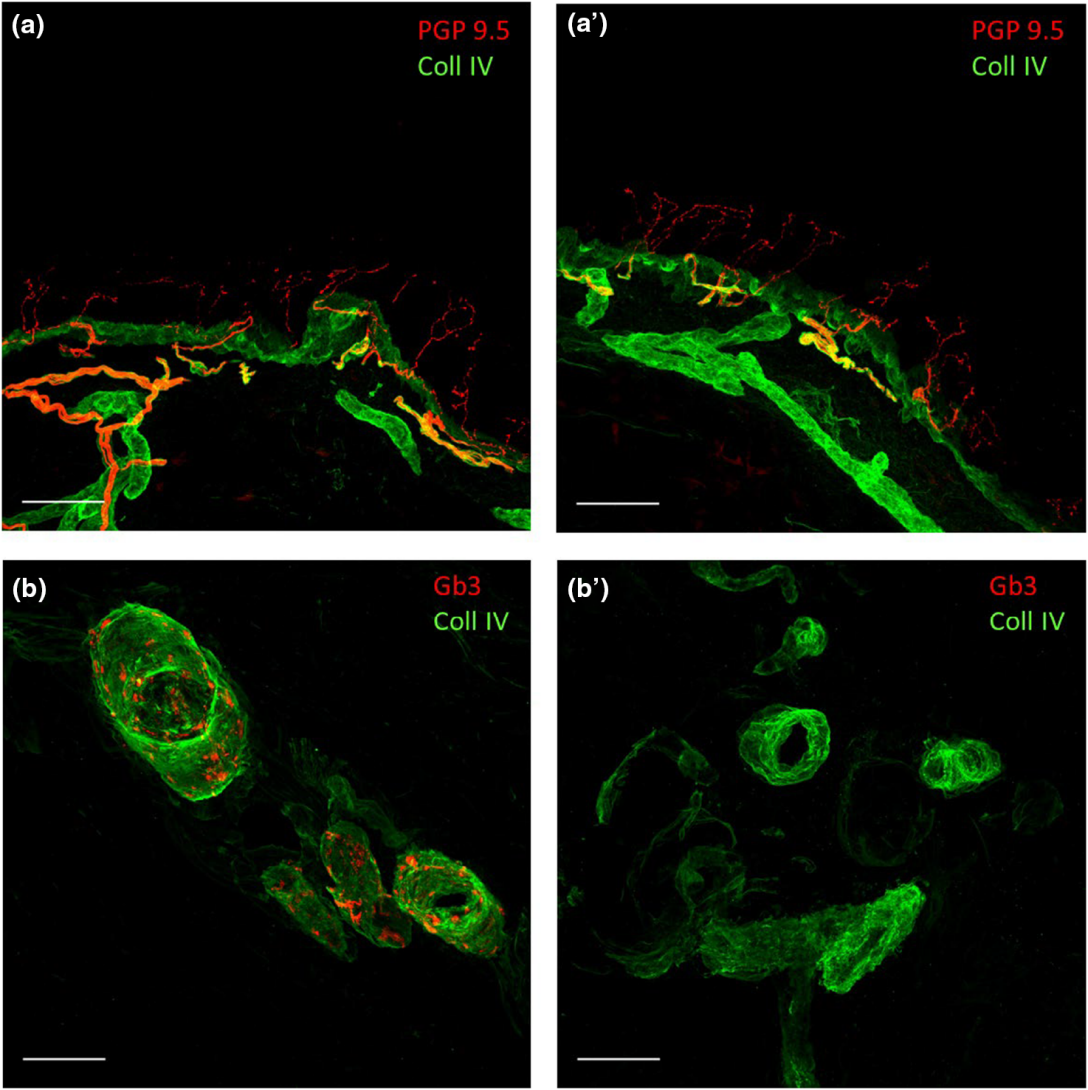
Epidermal innervation and Gb3 deposits in the analyzed patients and controls. Leg epidermal innervation and the study of Gb3 in skin vessels disclosed by confocal microscope (×40) in age‐matched controls (a and b) and the investigated patient (a' and b'). Epidermal innervation. Nerve fibers are reported in red and collagen staining in green. Abundant free‐ending PGP immunoreactive nociceptive fibers crossing the dermal–epidermal junction marked by collagen are evident in the epidermis of the patient (a') in a similar extent to a healthy control subject (a) supporting preserved small nerve fibers in the investigated patient. Skin Gb3 deposits. Skin arterioles showed no Gb3 deposits in the investigated patient (b') that are usually found in patients with classical GLA mutations. This is reported in (b) which illustrates the presence of Gb3 deposits (in red) in the wall of the arteriole in an age‐matched female FD patient carrying the classical mutation c.837G>C (Q279H). This patient presents a mild expression of the disease, as expected in females, with moderate hypertrophic cardiomyopathy and proteinuria (b). Bar = 50 μm.

Nevertheless, in order to definitively rule out the presence of FD in the index case and according to the patient's preferences, a kidney biopsy was performed to assess for potential renal involvement. The ultrastructural analysis of the kidney biopsy resulted in a completely normal without any “zebra bodies,” expression of substrate accumulation (Figure 2). The patient was further evaluated by a neurologist who performed a skin biopsy which also showed the absence of Gb3 inclusions (Figure 3).

Based on the comprehensive evaluation, the identified variant was definitively considered nonpathogenic, and the patient was found to be completely asymptomatic for the disease. Consequently, there was no indication to initiate any specific therapies for FD, and the patient was then discharged from our center.

## 3 DISCUSSION

The natural history of FD has greatly changed after the introduction of specific therapies such as Enzyme Replacement Therapy (ERT) or chaperone, which have successfully reduced morbidity and mortality. However, it is important to acknowledge that these therapies come with their own challenges: they are very expensive, burdensome, and demanding. Therefore, it is mandatory to define the real pathogenic role of a variant in FD, especially when encountering new variants. In adult male and female patients with new Fabry variants or missense GLA variants of uncertain significance (VUS), ERT should be considered depending on whether there is biochemical, histological, or imaging evidence of kidney, heart, or central nervous system (CNS) injury attributable to FD, even in the absence of other typical FD symptoms (Germain et al., 2022). Moreover, confirmation of the disease‐causing GLA variant is important to help establish the disease phenotype and rule out benign polymorphisms that may cause reduced levels of α‐Gal A activity, leading testing of at‐risk family members as well. Input of geneticists and physicians with extensive expertise in disease phenotypes, prevalence, inheritance, biomarkers, allele frequencies, disease‐specific databases, and literature greatly contributes to a more accurate interpretation of the pathogenicity of variants. Recently, it has been emphasized the importance of a high level of LysoGb3 as a diagnostic marker of pathogenicity in VUS (Ortiz et al., 2018). However, there are several reports of affected patients with VUS, where LysoGb3 was within the normal range despite the presence of deposits observed in kidney biopsy (Cerón‐Rodríguez et al., 2019). Alternatively, the identification of typical lysosomal inclusions in tissue biopsy specimens has been suggested as the gold standard of FD diagnosis (Schiffmann et al., 2016).

In our patient, all signs and symptoms of the disease were absent. However, due to the novelty of the variant, there was no indication of its pathogenicity. Although assessing males in suspected FD families can be helpful in identifying true disease manifestations, it is worth noting that the father had not been studied before. Given the normal enzyme activity of the father, it was reasonable to conclude that the variant was benign. Nevertheless, we decided to also perform a renal biopsy in the index case. The biopsy documented the complete absence of substrate accumulation in renal cells. This finding was confirmed by the result of a skin biopsy that was negative for deposits as well.

## 4 CONCLUSIONS

In conclusion, the recognition of new variants in FD, even in an asymptomatic patient, should prompt us to analyze the actual pathogenic role of the variant. The variant analysis in this study is limited to two members of the same family, therefore, further documentation may be useful to strengthen the data we described. This clinical report emphasizes the importance of conducting a comprehensive evaluation, which includes genetic testing and histological examination, to determine the pathogenicity of a variant and guide appropriate treatment decisions.

To this end, it is important to note that the histological evaluation represents the highest level of investigation capable of detecting the pathogenic role of a variant. For this reason, nephrologists should be encouraged to perform this evaluation, particularly in the presence of new variants of unknown significance, as this can provide crucial insights into the potential impact of the variant on organ function.

## AUTHOR CONTRIBUTIONS

Irene Capelli, Roberta Di Costanzo, Vincenzo Donadio, Renzo Mignani, and Gaetano La Manna: conceptualization, data collection, original draft preparation, and draft revision. Paola De Giovanni, Marcello Montevecchi, Davide Cerretani, Valeria Aiello, and Sarah Lerario: data collection and data interpretation. All authors reviewed the results and approved the final version of the manuscript.

## CONFLICT OF INTEREST STATEMENT

The authors declare no conflict of interest.

## ETHICAL STATEMENT

Ethical review and approval were waived for this study because, according to the local policy, informed consent is considered sufficient for reports of an observational nature concerning a limited number of patients. Informed consent was obtained from all subjects involved in the study.

## Data Availability

The data that support the findings of this study are available from the corresponding author upon reasonable request.
